# GRP78 recognizes EV-F 3D protein and activates NF-κB to repress virus replication by interacting with CHUK/IKBKB

**DOI:** 10.1128/jvi.00268-24

**Published:** 2024-05-22

**Authors:** Xiaoran Chang, Yidi Guo, Qun Zhang, Xuebo Zheng, Xuyuan Cui, Junying Hu, Zhiyuan Zhang, Fan Zhang, Xinping Wang

**Affiliations:** 1 State Key Laboratory for Diagnosis and Treatment of Severe Zoonotic Infectious Diseases, Key Laboratory for Zoonosis Research of the Ministry of Education, Institute of Zoonosis, and College of Veterinary Medicine, Jilin University, Changchun, China; Loyola University Chicago-Health Sciences Campus, Maywood, Illinois, USA

**Keywords:** GRP78, EV-F, 3D protein, NF-κB, CHUK, IKBKB, enterovirus replication

## Abstract

Enteroviruses are the causative agents associated with several human and animal diseases, posing a significant threat to human and animal health. As one of the host immune defense strategies, innate immunity plays a crucial role in defending against invading pathogens, where the host utilizes a variety of mechanisms to inhibit or eliminate the pathogen. Here, we report a new strategy for the host to repress enterovirus replication by the 78 kDa glucose-regulated protein (GRP78), also known as heat shock protein family A member 5 (HSPA5). The GRP78 recognizes the EV-encoded RNA-dependent RNA polymerases (RdRPs) 3D protein and interacts with the nuclear factor kappa B kinase complex (CHUK) and subunit beta gene (IKBKB) to facilitate the phosphorylation and nuclear translocation of NF-κB, which induces the production of inflammatory factors and leads to a broad inhibition of enterovirus replication. These findings demonstrate a new role of GRP78 in regulating host innate immunity in response to viral infection and provide new insights into the mechanism underlying enterovirus replication and NF-κB activation.

**IMPORTANCE:**

GRP78 is known as a molecular chaperone for protein folding and plays a critical role in maintaining protein folding and participating in cell proliferation, cell survival, apoptosis, and metabolism. However, the functions of GRP78 to participate in enterovirus genome replication and innate immune responses are rarely documented. In this study, we explored the functions of the EV-3D-interacting protein GRP78 and found that GRP78 inhibits enterovirus replication by activating NF-κB through binding to EV-F 3D and interacting with the NF-κB signaling molecules CHUK/IKBKB. This is the first report that GRP78 interacts with CHUK/IKBKB to activate the NF-κB signaling pathway, which leads to the expression of the proinflammatory cytokines and inhibition of enterovirus replication. These results demonstrate a unique mechanism of virus replication regulation by GRP78 and provide insights into the prevention and treatment of viral infections.

## INTRODUCTION

The innate immune system is a highly conserved signaling network crucial for protecting the host and eliminating the invading pathogen (1). One mechanism of innate immunity to control the virus infection is through the production of antiviral cytokines such as IL-6, IL-8, IL-1β, and TNF-α, where these cytokines either directly contain or trigger the signaling pathways involved in innate immune response against the invading pathogen. One such pathway is the nuclear factor-kappa B (NF-κB), which is considered a proinflammatory signaling pathway important in the host innate immune response. As a transcriptional factor, NF-κB functions as a vital regulator during RNA virus infection. A step critical for NF-κB activation is the phosphorylation of IκBα by IKKs (2), where the IKK complex has been demonstrated to be essential for the activation of canonical NF-κB signaling in response to various stimuli such as RNAs or DNAs derived from viruses. Phosphorylation of IκBα results in its degradation through the ubiquitin-proteasome system and subsequently leads to the release and nuclear translocation of the NF-κB transcription factor (3), triggering the expression of a variety of genes involved in eliciting innate or adaptive immunity and inflammation responses, which in turn limits viral replication (4–6). Simultaneously, a virus employs a strategy to evade the host immune response and maintains its replication via balancing NF-κB activation. EV71 enterovirus exploits a novel mechanism to inhibit IKKβ phosphorylation by recruiting PP1 and IKKβ to form a complex through 2C proteins, which ultimately results in the inhibition of the NF-κB signaling pathway (7). EV71 2C protein inhibits NF-κB activation via interaction with p65 and IKKβ (8).

The 78 kDa glucose-regulated protein (GRP78), also known as heat shock protein family A member 5 (HSPA5) or heavy chain-binding protein (BiP), is a member of the heat shock protein 70 kDa family (9, 10). As a master regulator of endoplasmic reticulum homeostasis and the molecular chaperones for protein folding, GRP78 is widely expressed in the endoplasmic reticulum (ER) and plays a critical role in maintaining protein folding and protein processing (11, 12), participating in cell proliferation, cell survival, apoptosis, and metabolism (13–15). In addition, GRP78 acts as a co-receptor for several viruses such as Coxsackievirus A9, Borna disease virus, and Zika virus (16–19). GRP78 contains an N-terminal ATPase domain, a C-terminal polypeptide-binding domain, and a highly helical C-terminal tail with a KDEL ER retention signal (20, 21). Previous studies have demonstrated the expression of GRP78 and NF-κB under oxidative stress and ER stress conditions (22, 23), and the interactions and regulation of NF-κB with other HSP members (24–27). For example, Hsp70 interacts with IκBα and p65 subunits (24), while Hsp27 and Hsp90 regulate TNF-α through IKK (28). Additionally, Hsp70 has been demonstrated as the key regulator of NF-κB activity (29, 30).

The *Enterovirus* genus within the family of *Picornaviridae* is taxonomically grouped into 12 *Enterovirus* (A–L) and 3 *Rhinovirus* (A–C) species (31). These viruses share similar characteristics that are non-enveloped and icosahedral with a positive-stranded RNA genome (32). Out of the 12 *Enterovirus* species, enterovirus E (EV-E) and enterovirus F (EV-F) viruses infect cattle (33–37), while enterovirus G (EV-G) viruses generally infect pigs (38–41). Recently, several enterovirus strains isolated from goats or sheep are also classified as EV-G (42–45).

The genome of enterovirus is approximately 7,100–7,450 nucleotides long, containing a single open reading frame that encodes a polyprotein precursor, which is subsequently cleaved into four structural proteins (VP1, VP2, VP3, and VP4) and seven non-structural proteins (2A, 2B, 2C, 3A, 3B, 3C, and 3D) (32). Among these proteins, the 3D functions as the RNA-dependent RNA polymerase (RdRp) crucial for viral genomic RNA synthesis. Previously, we characterized a novel caprine enterovirus strain SD-S67 as EV-F (46) and identified a 3D-interacting protein GRP78 using a co-immunoprecipitation (Co-IP) assay and mass spectrometry (unpublished data). We speculate that the 3D-interacting protein GRP78 might be involved in the regulation of the enterovirus replication. To test the hypothesis, we performed an in-depth study to explore how the newly identified enterovirus 3D-interacting protein (GRP78) affects enterovirus replication and the underlying mechanism. We found that GRP78 inhibits enterovirus replication by a new mechanism through interacting with the 3D protein, the NF-κB signaling proteins CHUK and IKBKB, to activate the NF-κB signaling pathway. These findings demonstrated a new role of GRP78 in the regulation of host innate immunity in response to NF-κB activation during viral infection and provide new insights into the mechanism underlying the replication of RNA viruses and the production of NF-κB upon viral infection.

## RESULTS

### Increased expression of GRP78 in cells upon enterovirus infection

**Fig 1:**
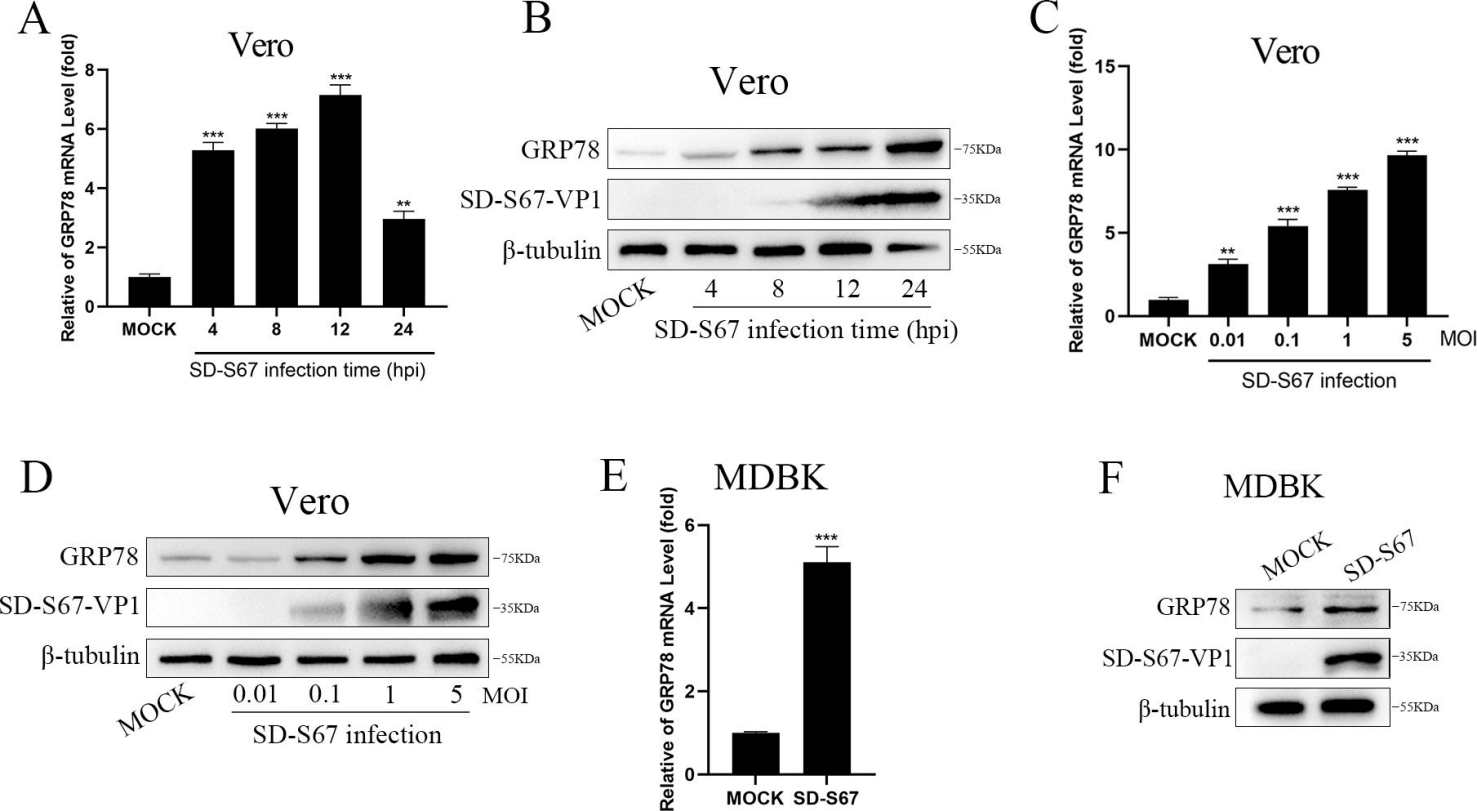
EV-F infection induces GRP78 expression. (**A and B**) Vero cells were mock-infected or infected by EV-F SD-S67 [multiplicity of Infection (MOI) of 1] for different time points. GRP78 and GAPDH mRNAs were determined by quantitative reverse transcription PCR (qRT-PCR) analyses (**A**). GRP78, SD-S67-VP1, and β-tubulin proteins were detected by Western blot analyses with corresponding antibodies (**B**). (**C and D**) Vero cells were mock-infected or infected by EV-F SD-S67 for 12 h using different MOIs. GRP78 and GAPDH mRNAs were determined by qRT-PCR analyses (**C**). GRP78, SD-S67-VP1, and β-tubulin proteins were detected by Western blot analyses with corresponding antibodies (**D**). (**E and F**) GRP78 and GAPDH mRNA expression in MDBK cells mock-infected or infected by EV-F SD-S67 (MOI of 1) for 12 h was determined by qRT-PCR analyses (**E**). GRP78, SD-S67-VP1, and β-tubulin protein were detected by Western blot analyses with corresponding antibodies (**F**). Data are presented as the mean ± SD values. *P*-values <0.05 (*), <0.01 (**), and <0.001 (***) were considered statistically significant and highly significant, respectively.

Preliminary proteomics studies in our laboratory demonstrated a significant increase of GRP78 protein levels in Vero cells infected by enterovirus. To substantiate that the increased expression of GRP78 was indeed induced by enterovirus infection, Vero cells infected by EV-F SD-S67 were used to assay the expression of GRP78. The results demonstrated that both GRP78 mRNA (Fig. 1A) and protein (Fig. 1B) were substantially induced by enterovirus at 4 h post-infection (hpi) compared with the mock-infected Vero cells. In addition, GRP78 mRNA (Fig. 1C) and GRP78 protein (Fig. 1D) were elevated in a dose-dependent manner in cells infected by different dosages of enterovirus. Similar results were also observed in MDBK cells infected by EV-F, where GRP78 mRNA (Fig. 1E) and protein (Fig. 1F) levels were significantly induced. The above results demonstrated that the expression of GRP78 is induced by EV-F enterovirus and is in a dosage-dependent fashion and cell-independent manner.

### Inhibition of enterovirus replication by GRP78

**Fig 2:**
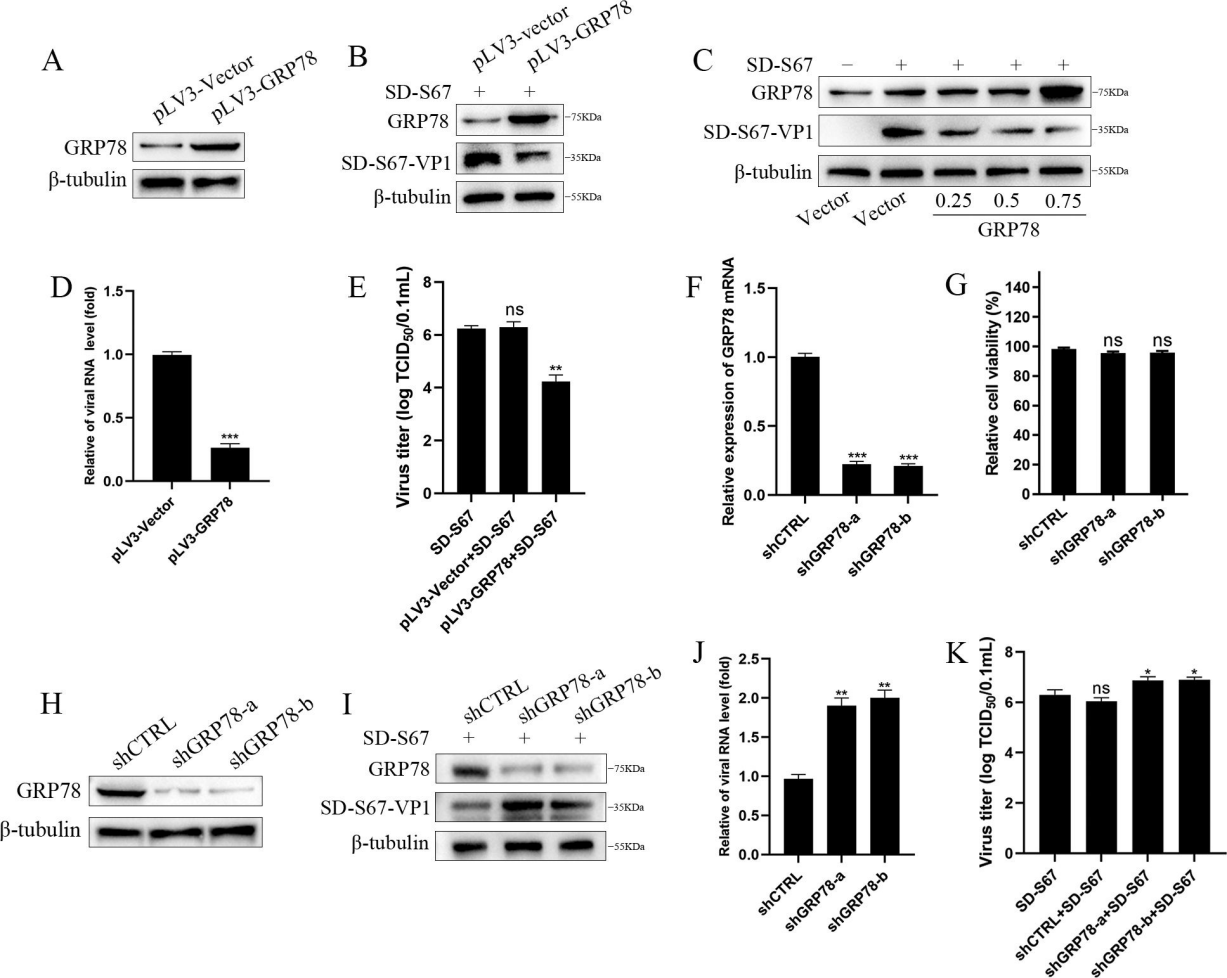
GRP78 inhibits EV-F replication. (**A**) GRP78 and β-tubulin proteins were detected by Western blot analyses in 293T cells transfected with pLV3-Vector (0.5 µg) or pLV3-GRP78 (0.5 µg) for 48 h. (**B–E**) 293T cells transfected with pLV3-Vector (0.5 µg) or pLV3-GRP78 (0.5 µg) or different concentrations of pLV3-GRP78 (**C**) for 24 h were infected with SD-S67 for 12 h. GRP78, SD-S67-VP1, and β-tubulin proteins were detected by Western blot analyses with corresponding antibodies (**B and C**). SD-S67-VP1 and GAPDH RNA were determined by qRT-PCR analyses (**D**). Viral titers at 48 hpi were determined and expressed as TCID_50_/0.1 mL (**E**). (**F and H**) 293T cells were co-transfected with pLVX-shGRP78 (**A and B**), paPAX2, and pMD2G or pLVX-shRNA as negative control for 72 h. The supernatants used to infect cells for 48 h. GRP78 and GAPDH RNA were determined by qRT-PCR analyses (**F**). Cell viability for pLVX-shGRP78-a and pLVX-shGRP78-b was detected using CCK-F kit (**G**). GRP78 and β-tubulin protein expression was detected by Western blot analyses (**H**). (**I–K**) 293T cells stably transduced with control or shGRP78 were infected with EV-F SD-S67 (MOI of 1) for 12 h. GRP78 or viral VP1 expression was determined by Western blot (**I**) or qRT-PCR (**J**). Viral titers at 48 hpi were determined and expressed as TCID_50_/0.1 mL (**K**). Results are representative of three independent experiments. Data are presented as mean ± SD values. *P*-values <0.05 (*), <0.01 (**), and <0.001 (***) were considered statistically significant and highly significant, respectively. ns, not significant.

To determine the effect of GRP78 on enterovirus replication, the generated GRP78 expression plasmid was overexpressed in 293T cells since it is relatively easy for transfection and also susceptible to EV infection. Exogenous GRP78 was shown to express in 293T cells compared with the pLV3-vector control (Fig. 2A). After infection by EV-F for 12 h, the expression of viral protein and mRNA and 50% tissue culture infectious dose (TCID_50_) titers were significantly lower in 293T cells overexpressed with GRP78 in relation to these in control cells (Fig. 2B, D, and E), suggesting that GRP78 expression inhibits virus replication. VP1 protein expression was downregulated by GRP78 in a dose-dependent fashion (Fig. 2C).

To further confirm the inhibition effect of GRP78 on EV replication, GRP78 knocked down using GRP78-specific shRNAs (shGRP78-a, shGRP78-b) in 293T cells were verified, with shCTRL as vector control (Fig. 2F and H). A cell viability assay showed that the knockdown of GRP78 had no significant effect on cell viability (Fig. 2G). The 293T cell lines with GRP78 knockdown were then infected by EV-F before harvesting for virus titer and viral replication assessment. The knockdown of GRP78 in 293T cells increased the virus titer and VP1 expression level in relation to the control cells (Fig. 2I through K). Collectively, these results demonstrated the inhibition effect of GRP78 on EV-F replication.

### Interaction of 3D protein with GRP78

**Fig 3:**
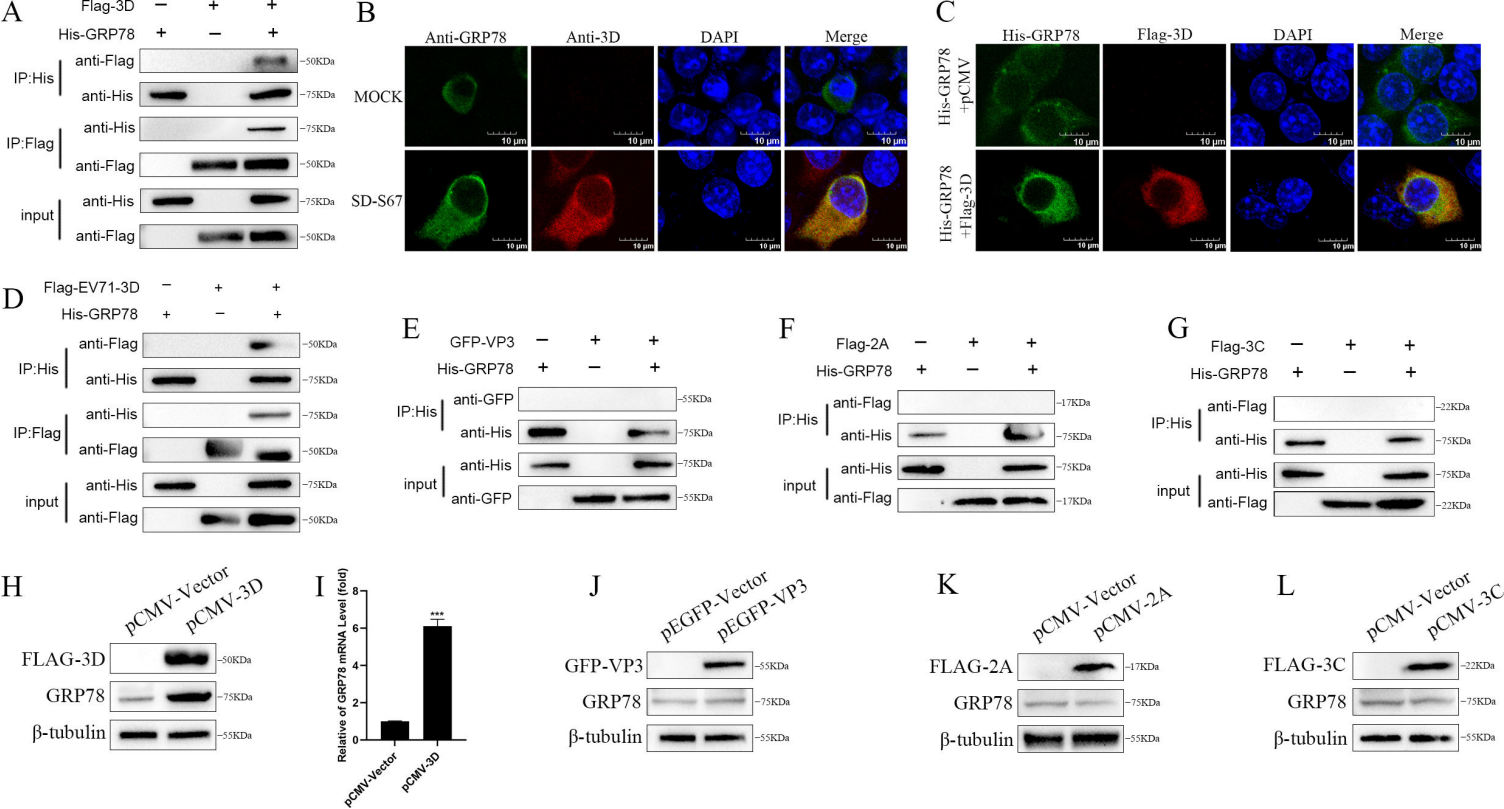
3D protein interacts with GRP78. (**A**) Interactions of 3D with GRP78 were detected in 293T cells co-transfected with Flag-tagged 3D (2.5 µg) and His-tagged GRP78 (2.5 µg) by immunoprecipitation and immunoblotting using anti-Flag or anti-His antibody. (**B**) Co-localization of 3D (red) with GRP78 (green) was analyzed by confocal microscopy using FV3000 software in 293T cells mock-infected or infected with EV-F SD-S67 (MOI of 1) for 36 h. Cell nuclei were stained with 4',6-diamidino-2-phenylindole (DAPI) (blue). Scale bar, 10 µm. (**C**) The co-localization of Flag-tagged 3D (0.5 µg) (red) and His-tagged GRP78 (0.5 µg) (green) was analyzed in 293T cells co-transfected with Flag-3D and His-GRP78 plasmids for 48 h. The 293 cells co-transfected of His-GRP78 with pCMV-vector were used as negative control. Cell nuclei were stained in DAPI (blue). Scale bar, 10 µm. (**D**) Interactions of EV71-3D with GRP78 were detected in 293T cells co-transfected with Flag-tagged EV71-3D (2.5 µg) and His-tagged GRP78 (2.5 µg) by immunoprecipitation and immunoblotting using anti-Flag or anti-His antibody. (**E–G**) 293T cells co-transfected with GFP-tagged VP3 (2.5 µg) and His-tagged GRP78 (2.5 µg) (**E**), Flag-tagged 2A (2.5 µg) and His-tagged GRP78 (2.5 µg) (**F**), and Flag-tagged 3C and His-tagged GRP78 (**G**) were immunoprecipitated using anti-His antibody, and interactions between VP3 and GRP78 (**E**), 2A and GRP78 (**F**), and 3C and GRP78 (**G**) were detected with immunoblotting, respectively. (**H–L**) 293T cells were transfected with pCMV-Tag2B-3D (0.5 µg) (**H and I**), pEGFP-N1-VP3 (0.5 µg) (**J**), pCMV-Tag2B-2A (0.5 µg) (**K**), and pCMV-Tag2B-3C (0.5 µg) (**L**), respectively, for 48 h. GRP78 mRNA was determined by qRT-PCR analyses (**I**). GRP78, Flag-3D, GFP-VP3, Flag-2A, Flag-3C, and β-tubulin proteins were detected by Western blot analyses with corresponding antibodies (**H, J–L**). The experiment was repeated three times with similar results.

Previously, we performed the co-immunoprecipitation with 3D antibody to investigate the 3D-interacting proteins. After mass spectrometry analyses, GRP78 was identified as one of the potential 3D-interacting proteins. To determine if 3D protein indeed interacts with GRP78, 293T cells co-transfected with Flag-tagged 3D and His-tagged GRP78 plasmids were harvested for Co-IP assays. As shown in Fig. 3A, when exogenous Flag-tagged 3D proteins or His-tagged GRP78 were used as bait proteins, they had the ability to precipitate each other compared with the controls. The interaction between 3D protein and GRP78 was confirmed further by confocal microscopy and shown to co-localize in the cytoplasm (Fig. 3B and C). Similarly, Co-IP was performed using Flag-tagged EV71-3D protein and His-tagged GRP78. As shown in Fig. 3D, when exogenous Flag-tagged EV71-3D protein or His-tagged GRP78 was used as bait proteins, they had the ability to precipitate each other compared with the controls. These results demonstrated that 3D protein indeed interacts with GRP78. No interactions were detected between the EV-encoded protein VP3, 2A, and 3C with GRP78 by the Co-IP assay (Fig. 3E through G).

To investigate whether VP3, 2A, 3C, and 3D proteins had any effect on GRP78 expression, the expression plasmids for GFP-tagged VP3, Flag-tagged 2A, Flag-tagged 3C, and Flag-tagged 3D were used to transfect the 293T cells. The expression of GRP78 protein (Fig. 3H) and mRNA (Fig. 3I) was significantly elevated in cells transfected with Flag-tagged 3D, while it had no significant change in cells transfected with GFP-tagged VP3, Flag-tagged 2A, and Flag-tagged 3C, plasmids in comparison with vector (Fig. 3J through L), indicating that 3D protein is likely related to the increase of GRP78 expression.

### Activation of NF-κB expression by EV infection

**Fig 4:**
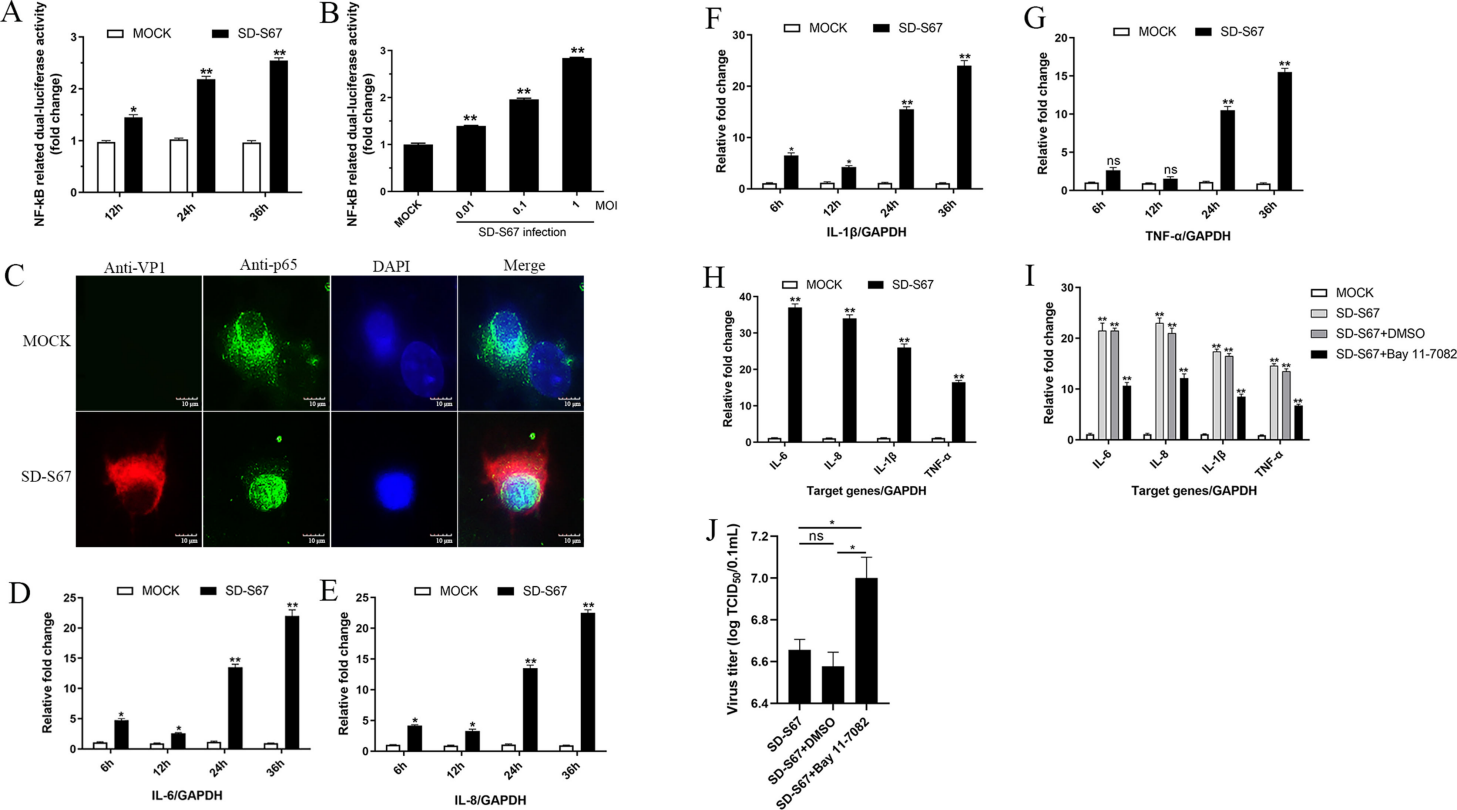
EV-F infection activated the NF-κB pathway. (**A**) Effects of EV infection time on the NF-κB activation. 293T cells were co-transfected with NF-κB-Luc reporter plasmid (0.2 µg) and pRL-SV40 plasmid (0.05 µg). Twenty-four hours post-transfection, the cells mock-infected or infected with EV-F SD-S67 (MOI of 1) for different time points (12, 24, and 36 hpi) were harvested for luciferase reporter gene assays. (**B**) Dose effect of EV infection on the activation of NF-κB. Cells mock-infected or infected by different MOIs (0.01, 0.1, and 1) of EV-F SD-S67 were harvested at 36 h post-infection for luciferase reporter gene assays. (**C**) Subcellular localizations of VP1 (red) and p65 (green) were analyzed in 293T cells mock-infected or infected by EV-F SD-S67 (MOI of 1) for 36 h using FV3000 software. Nuclei were stained with DAPI (blue). Scale bar, 10 µm. (**D–H**) Expression levels of mRNA for IL-6, IL-8, IL-1β, and TNF-α were measured by qPCR in 293T cells and Raw264.7 cells mock-infected or infected with EV-F SD-S67 (MOI of 1). GAPDH was used as internal reference genes. (**I and J**) Inhibition of the NF-κB activation induced by EV using Bay 11-7082. Bay 11-7082 was added to 293T cells infected with EV-F SD-S67 (MOI of 1) at a final concentration of 5 μΜ at 1 h post-infection. Cells treated with Dimethyl sulfoxide (DMSO) served as negative controls. The mRNA expression levels of IL-6, IL-8, IL-1β, and TNF-α were measured at 36 h post-infection via qPCR. GAPDH was used as internal reference genes (**I**). Viral titers at 48 hpi were determined and expressed as TCID_50_/0.1 mL (**J**). Results are representative of three independent experiments. Data are presented as means ± SD. *P*-values <0.05 (*) and <0.01 (**) were considered to be statistically significant.

To determine the effects of EV infection on the NF-κB signaling pathway, 293T cells were transiently transfected with the pNF-κB-Luc reporter plasmid along with pRL-SV40. Twelve hours post-transfection, the cells were infected by EV-F, with the mock-infected cells as control. After infection, cells collected at different time points were used to assay the luciferase activity. The luciferase activity of EV-infected cells was significantly higher than that of the mock-infected group (Fig. 4A), suggesting that the virus infection activates/increases the NF-κB expression. To evaluate the dose effect of EV infection on NF-κB activity, 293T cells transfected with pNF-κB-Luc and pRL-SV40 were infected with EV-F using different MOIs. EV-F infection enhanced the NF-κB luciferase activity in a dose-dependent manner (Fig. 4B). To further confirm that EV-F infection indeed activates the NF-κB signaling pathway, p65 expression was monitored in 293T cells infected by EV using fluorescence microscopy. P65 protein expression was mainly accumulated in the nucleus in the EV-infected cells, where it was located mainly in the cytoplasm of mock-infected cells (Fig. 4C). These results demonstrated the translocation of p65 and confirmed the activation of the NF-κB signaling pathway following EV infection.

Since the NF-κB signaling pathway was activated in 293T cells after EV infection, the expression of NF-κB-regulated proinflammatory genes in EV-infected cells was examined using real-time PCR (qPCR) with GAPDH as the internal reference gene. The mRNA levels of IL-6, IL-8, IL-1β, and TNF-α were significantly elevated following EV infection (Fig. 4D through G). Moreover, the increased mRNA expression of IL-6, IL-8, IL-1β, and TNF-α was also detected in Raw264.7 cells infected by EV (Fig. 4H), suggesting that the inflammation induced by EV is cell type-independent. To further ensure that the proinflammatory gene expression was associated with the NF-κB signaling pathway, the cells were treated with Bay 11-7082, an inhibitor specifically blocking NF-κB activation. Results showed a dramatically decreased expression of proinflammatory genes in EV-infected cells (Fig. 4I). The viral titer in cells treated with Bay 11-7082 was increased compared to the DMSO-treated group (Fig. 4J). These results demonstrated the NF-κB activation and the inflammatory responses induced by EV infection.

### Activation of NF-κB by 3D during the EV infection

**Fig 5:**
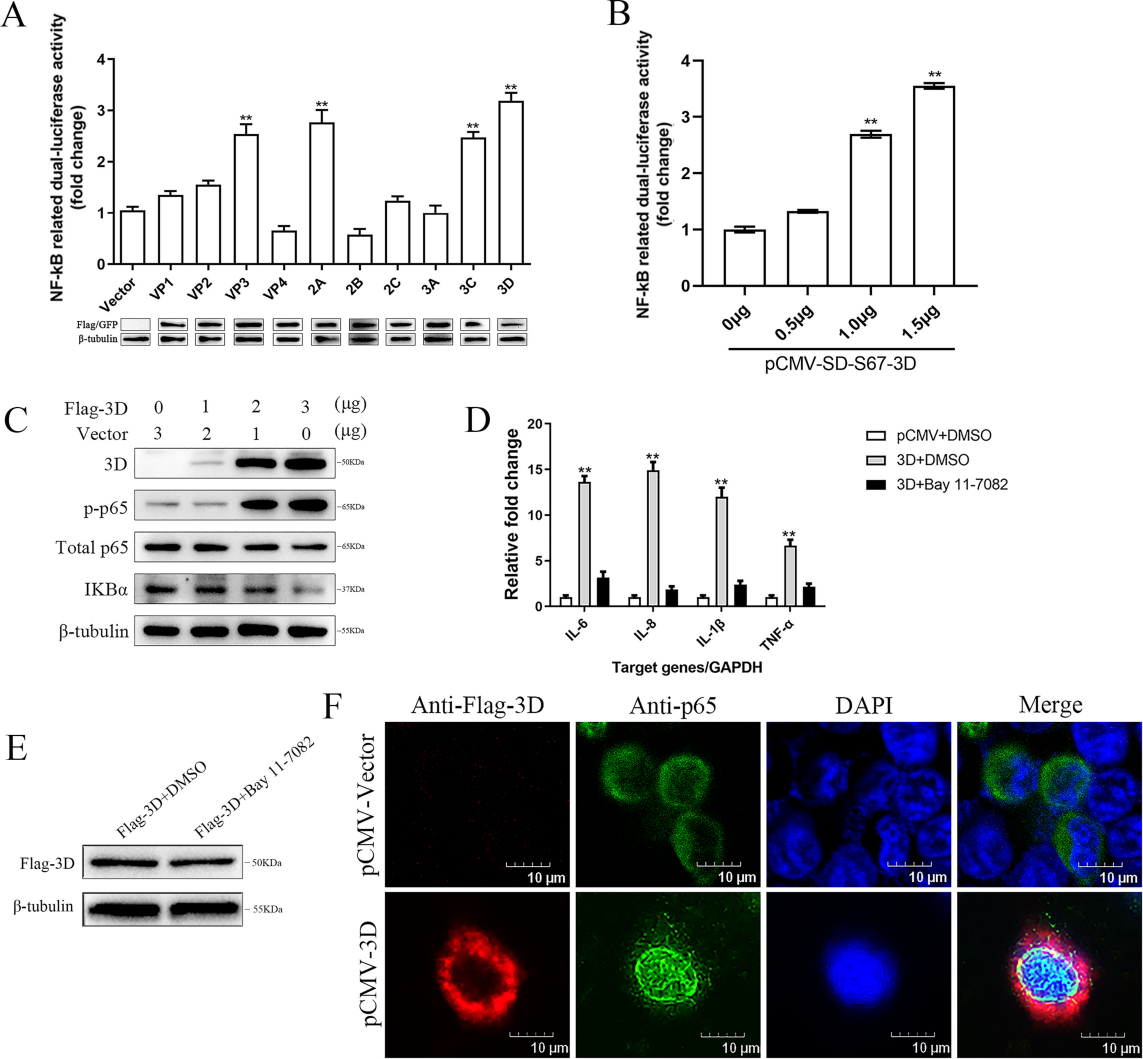
EV-F 3D protein activated NF-κB pathway. (**A**) EV-encoded proteins involved in the activation of NF-κB. 293T cells were co-transfected with pNF-κB-Luc (0.2 µg), pRL-SV40 (0.05 µg), and the expression plasmids harboring different EV SD-S67 genes (VP1, VP2, VP3, VP4, 2A, 2B, 2C, 3A, 3C, and 3D) (1 µg). Forty-eight hours post-transfection, cell extracts were prepared for luciferase reporter gene assays. The expression of EV-encoded proteins is shown below the bar chart. (**B**) Increasing quantities of 3D expression plasmids (0, 0.5, 1, and 1.5 µg) were co-transfected with pNF-κB-Luc and pRL-SV40 into 293T cells. Cells were harvested and analyzed by luciferase activity measurement at 48 h post-transfection. (**C**) 293T cells were transfected with 0, 1, 2, or 3 µg of Flag-tagged 3D for 48 h. Flag-3D, p-p65, p65, IκBα, and β-tubulin protein expression was detected by Western blot analyses. (**D**) 293T cells were transfected with Flag-tagged 3D (1 µg) or the pCMV vector (1 µg), respectively, for 12 h; Bay 11-7082 was added to the culture media to a final concentration of 5 µM for 24 h. The mRNA expression levels of IL-6, IL-8, IL-1β, and TNF-α were measured via qPCR. GAPDH was used as internal reference gene. Cells transfected with pCMV plasmid or treated with DMSO served as negative controls. (**E**) 293T cells were transfected with Flag-tagged 3D (1 µg) for 12 h; Bay 11-7082 was added to the culture media to a final concentration of 5 µM for 24 h. The Flag-3D expression level was measured via Western blot. Cells treated with DMSO served as negative controls. (**F**) Flag-3D (1 µg) was transiently expressed in 293T cells for 48 h. The subcellular localizations of Flag-tagged 3D (red), p65 (green), and nucleus marker DAPI (blue) were analyzed using FV3000 software. Scale bar, 10 µm. Results are representative of three independent experiments. Data are presented as mean ± SD values. *P*-values <0.05 (*) and <0.01 (**) were considered statistically significant and highly significant, respectively.

To identify the EV proteins involved in the activation of the NF-κB signaling pathway, the generated pCMV-Tag2B or pEGFP-N1 expression plasmids expressing different EV-encoded proteins were cotransfected with pNF-κB-Luc reporter plasmid in 293T cells with corresponding mock controls. As shown in Fig. 5A, the luciferase activities were all increased to a varied extent in cells transfected with plasmids expressing EV-encoded proteins with the exception of VP4 and 2B. It is worthwhile to note that 3D exhibited the strongest activation capability.

To confirm the activation of the NF-κB signaling pathway by 3D protein, an increasing amount of pCMV-Tag2B-3D-expressing plasmids were co-transfected with pNF-κB-Luc reporter plasmid to 293T cells. The luciferase activity levels increased gradually in the transfected 293T cells correlated with the increased expression of 3D (Fig. 5B).

The degradation of IκBα following its phosphorylation by the IKK complex and nuclear translocation of p65 is considered to be the hallmarks of NF-κB signaling pathway activation (47). To further validate the activation of NF-κB by 3D protein, 293T cells were transfected with increasing amounts of pCMV-Tag2B-3D, and the same set of samples were assayed to investigate the expression levels of IκBα, p65, and p-p65 using the Western blot assay. The results showed that the expression of 3D had no significant effects on the total amount of p65. However, the level of p-p65 increased markedly and the level of IκBα decreased dramatically (Fig. 5C).

To determine whether 3D contributed to the enhancement of NF-κB-regulated proinflammatory gene expression induced by EV infection, 293T cells transfected with the pCMV-Tag2B-3D were treated with NF-κB-specific inhibitor (Bay 11-7082) at 12 h post-transfection. The results showed that 3D enhanced the expression of NF-κB-regulated proinflammatory genes IL-6, IL-8, IL-1β, and TNF-α. However, in the NF-κB-specific inhibitor-treated group, 3D exhibited a reduced ability to upregulate the expression of these genes compared to that observed with the DMSO-treated group (Fig. 5D). The NF-κB inhibitor Bay 11-7082 did not affect the expression of 3D protein (Fig. 5E). These findings suggest that 3D played a critical role in stimulating proinflammatory gene expression, which was induced via the NF-κB signaling pathway during EV infection.

To determine the nuclear translocation of NF-κB, p65 was detected in 293T cells expressing Flag-tagged 3D fusion protein using indirect immunofluorescence (IFA). The IFA data showed that endogenous p65 was indeed translocated to the nucleus in cells with 3D protein expressed, while it was accumulated in the cytoplasm of the cells transfected with the empty vector (Fig. 5F). These results demonstrated that 3D protein activated the NF-κB signaling pathway during EV infection.

### GRP78 is involved in the NF-κB activation

**Fig 6:**
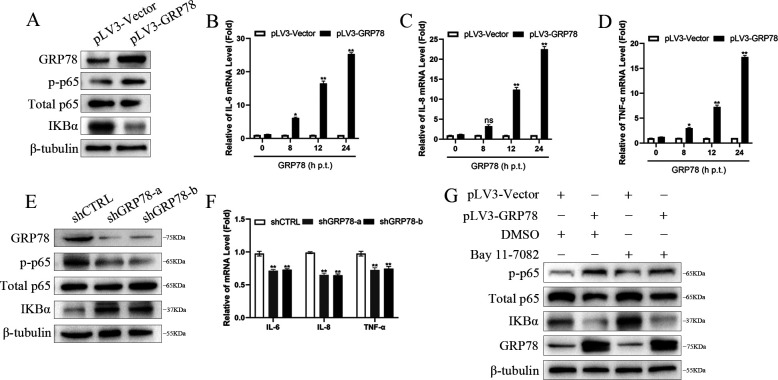
GRP78 activated NF-κB pathway. (**A**) GRP78, p-p65, total p65, IKBα, and β-tubulin proteins were detected in 293T cells transfected with pLV3-GRP78 (0.5 µg) or pLV3-Vector (0.5 µg) for 48 h with corresponding antibodies using Western blot assay. (**B–D**), IL-6, IL-8, TNF-α, and GAPDH mRNA expression was determined by qRT-PCR in 293T cells transfected with pLV3-GRP78 (0.5 µg) for different time points (0, 8, 12, and 24 h). (**E and F**) GRP78, p-p65, total p65, IKBα, and β-tubulin protein expression was examined in 293T cells with GRP78 knocked down by shGRP78, using corresponding antibodies by Western blot assay (**E**). IL-6, IL-8, TNF-α, and GAPDH mRNA expression from the same set of samples (**E**) was determined by qRT-PCR (**F**). (**G**) 293T cells were transfected with pLV3-GRP78 (0.5 µg) or pLV3-Vector (0.5 µg) for 24 h, followed by treatment with Bay 11-7082 (5 µM) for another 24 h. The cells were then subjected to Western blotting with antibodies against p-p65, p65, IKBα, GRP78, and β-tubulin. Results are representative of three independent experiments. Data are presented as mean ± SD values. *P*-values <0.05 (*) and <0.01 (**) were considered statistically significant and highly significant, respectively.

Previous studies by other researchers found that virus infection activates the expression of NF-κB and GRP78 via interacting with the protein members of the NF-κB family (24, 25). Since GRP78 was discovered to inhibit EV replication after its activation triggered by EV infection, it is reasonable to speculate that the inhibition of EV replication by GRP78 might be associated with the activation of NF-κB signaling molecules. The role of GRP78 in the regulation of NF-κB was determined in 293T cells transfected with pLV3-GRP78, and Western blot analyses were used to investigate the expression levels of IκBα and phosphorylation of p65 in cell lysates. The results showed an increased level of p-p65 and a decreased level of IκBα (Fig. 6A). Simultaneously, dramatic increases of the mRNA levels for IL-6, IL-8, and TNF-α were demonstrated in 293T cells transfected with pLV3-GRP78 for 8, 12, or 24 h. Moreover, the mRNA expression of these cytokines induced by GRP78 expression was also observed in a time-dependent manner (Fig. 6B through D). Additionally, the knockdown of GRP78 in 293T cells remarkedly reduced the level of p-p65 and increased the level of IκBα (Fig. 6E). The mRNA levels for IL-6, IL-8, and TNF-α in 293T cells with GRP78 knockdown were decreased (Fig. 6F). Furthermore, treatment of 293T cells transfected with pLV3-GRP78 using Bay 11-7082 (5 µM) significantly inhibited the activation of NF-κB signaling, as demonstrated by the decreased levels of p-p65 (Fig. 6G). These results suggest that GRP78 is involved in activating NF-κB signaling.

### GRP78 interacts with CHUK/IKBKB to promote NF-κB activation

**Fig 7:**
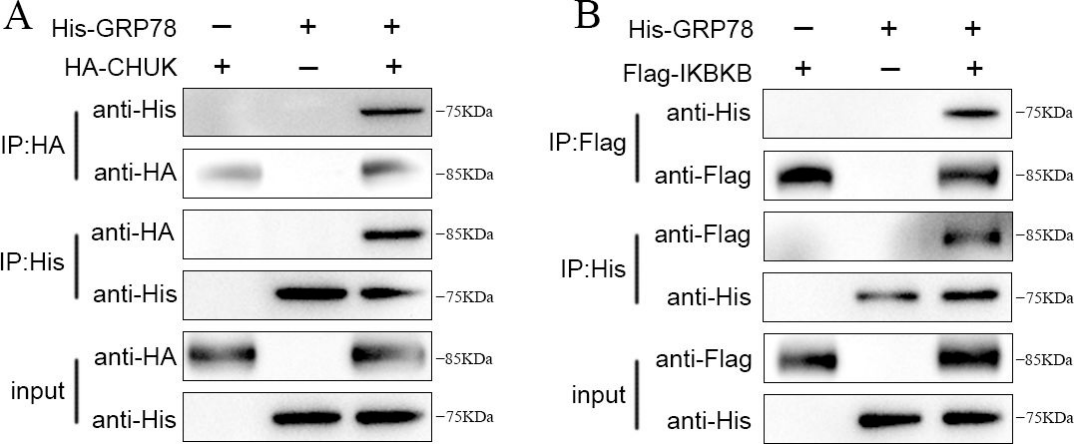
GRP78 interacts with CHUK and IKBKB. (**A**) Interactions of GRP78 with CHUK. 293T cells co-transfected with His-tagged GRP78 (2.5 µg) and HA-tagged CHUK (2.5 µg) were used for immunoprecipitation using anti-His or anti-HA antibody. Interactions between GRP78 and CHUK were detected with immunoblotting. (**B**) Interactions of GRP78 with IKBKB detected by Co-IP. 293T cells co-transfected with His-tagged GRP78 (2.5 µg) and Flag-tagged IKBKB (2.5 µg) were immunoprecipitated using anti-His or anti-Flag antibody. Interactions between GRP78 and IKBKB were detected with immunoblotting.

The above results showed that 3D protein activates the NF-κB signaling pathway and interacts with GRP78. To demonstrate the interaction between GRP78 and the proteins of the NF-κB signaling pathway, the His-tagged GRP78 was co-transfected with either HA-tagged CHUK or Flag-tagged IKBKB plasmid into 293T cells for Co-IP assays. GRP78 and CHUK were both successfully expressed as soluble proteins detected with rabbit anti-His or mouse anti-HA polyclonal antibody (pAb) (Fig. 7A). Subsequently, anti-His or anti-HA magnetic beads were exploited for exogenous Co-IP analysis. As shown in Fig. 7A, when exogenous proteins His-tagged GRP78 or HA-tagged CHUK were used as bait proteins, they captured each other, which was not observed in the control groups. The results provide evidences that GRP78 specifically interacts with CHUK. Similarly, the recombinant GRP78 and IKBKB were both expressed as soluble proteins and interacted with each other as detected using rabbit anti-His or rabbit anti-Flag pAb (Fig. 7B), demonstrating that GRP78 specifically interacts with IKBKB.

Taken together, the above results demonstrated the interactions of GRP78 with NF-κB signaling proteins CHUK and IKBKB.

### Inhibition of virus replication by GRP78 through activating NF-κB

**Fig 8:**
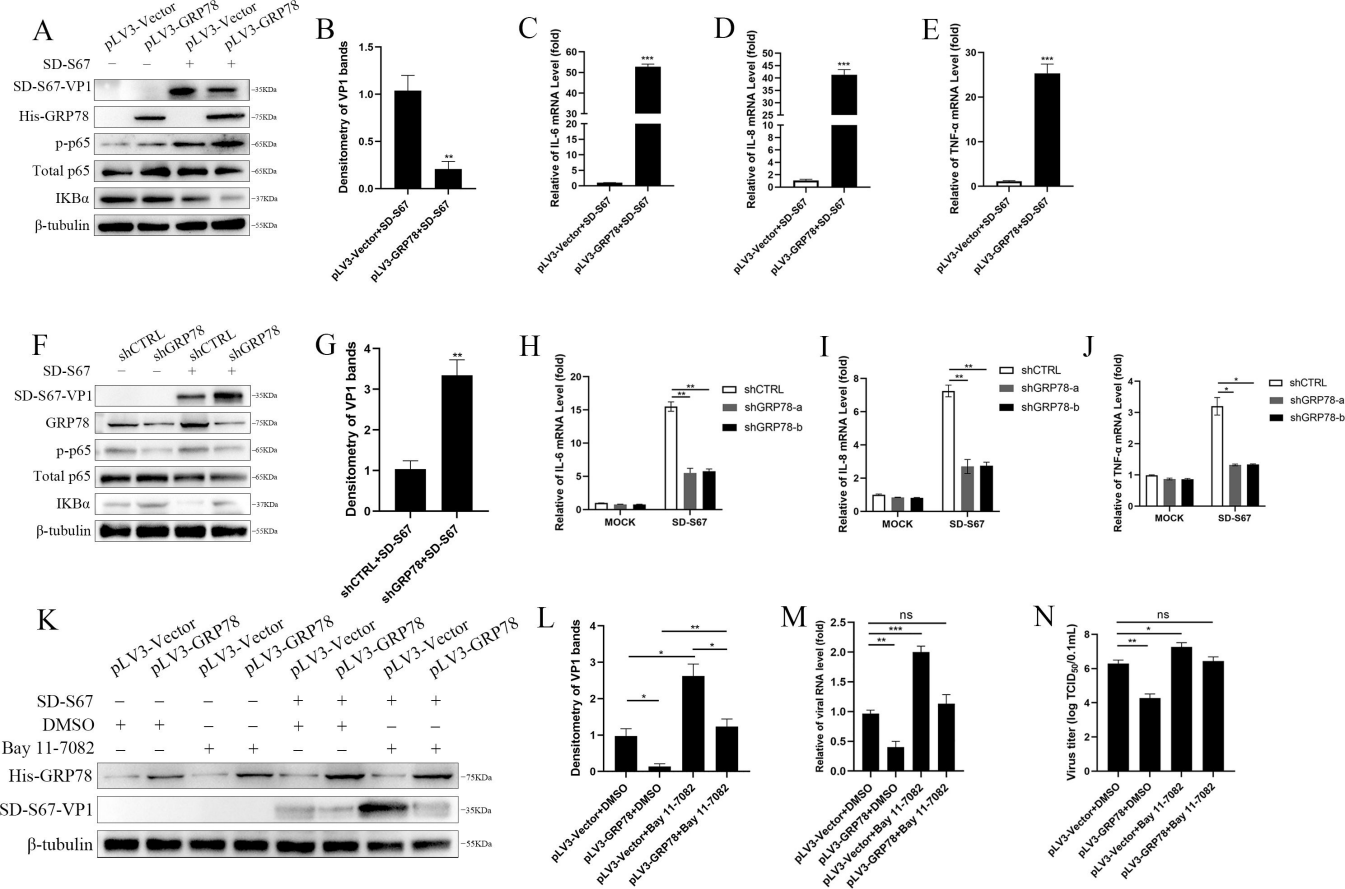
GRP78 regulates the nuclear translocation of NF-κB during viral infection. (**A–E**) 293T cells were transfected with pLV3-Vector (0.5 µg) or pLV3-GRP78 (0.5 µg) for 24 h before infection with SD-S67 (MOI of 1) for 24 h. Cell lysates were subjected to Western blot analyses with the p-p65, p65, IKBα, GRP78, and β-tubulin antibodies (**A**). Band of the VP1 expression from (**A**) was analyzed by scanning densitometry and normalized to the amount of loading control, β-tubulin. The fold changes are shown relative to the levels of the pLV3-Vector-infected cells. The data are presented as the mean values of three independent experiments (**B**). Total RNAs extracted from the cells were used to determine the expression of IL-6 (**C**), IL-8 (**D**), and TNF-α (**E**) using qRT-PCR with GAPDH as internal control. (**F–J**) 293T cells knocked down with shGRP78 or shCTRL were infected with EV-F SD-S67 (MOI of 1) for 24 h. Cell lysates were prepared and p-p65, p65, IKBα, GRP78, and β-tubulin proteins were detected by Western blot analyses (**F**). Band of the VP1 expression from (**F**) was analyzed by scanning densitometry and normalized to the amount of loading control, β-tubulin. The fold changes are shown relative to the levels of the shCTRL-infected cells. The data are presented as the mean values of three independent experiments (**G**). IL-6 (**H**), IL-8 (**I**), TNF-α (**J**), and GAPDH mRNA expression was examined by qRT-PCR using the total RNAs isolated from the cells. (**K–N**) 293T cells transfected with pLV3-GRP78 (0.5 µg) or pLV3-Vector (0.5 µg) for 12 h were treated using Bay 11-7082 (5 µM) for another 12 h, followed by infection of the cells with SD-S67 (MOI of 1) for 24 h. Cell lysates were subjected to Western blot analyses with the SD-S67 VP1, GRP78, and β-tubulin antibodies (**K**). Band of the VP1 expression from (**K**) was analyzed by scanning densitometry and normalized to the amount of loading control, β-tubulin. The fold changes are shown relative to the levels of the pLV3-Vector-infected and DMSO-treated cells. The data are presented as the mean values of three independent experiments (**L**). Total RNAs extracted from the cells were used to examine the SD-S67-VP1 and GAPDH mRNA expression by qRT-PCR analyses (**M**). Viral titers from the cells infected at 48 hpi were determined and expressed as TCID_50_/0.1 mL (**N**). Results are representative of three independent experiments. Data are presented as mean ± SD values. *P*-values <0.05 (*), <0.01 (**), and <0.001 (***) were considered statistically significant and highly significant, respectively.

To elucidate the mechanism underlying the inhibition of virus replication by GRP78, 293T cells transfected with pLV3-GRP78 were infected with EV. Western blot analyses showed that the expression of GRP78 enhanced p-p65 and decreased IκBα expression, while it also decreased the expression level of SD-S67 VP1 protein compared to vector control (Fig. 8A). Densitometric analysis showed that the protein levels of SD-S67-VP1 in virus-infected cells with GRP78 overexpression were lower than those in the empty vector group cells (Fig. 8B). Simultaneously, the expression of GRP78 significantly induced the mRNA levels of IL-6, IL-8, and TNF-α (Fig. 8C through E). In contrast, the knockdown of GRP78 in the 293T cells reduced p-p65 and increased IκBα expression (Fig. 8F), resulting in the increased expression of SD-S67 VP1 protein (Fig. 8F). Densitometric analysis showed that the protein levels of SD-S67-VP1 in virus-infected cells with GRP78 knockdown were higher than those in the control cells (Fig. 8G). Moreover, the knockdown of GRP78 reduced the mRNA levels of IL-6, IL-8, and TNF-α (Fig. 8H through J). Furthermore, 293T cells overexpressing GRP78 treated with NF-κB-specific inhibitor (Bay 11-7082) after infection by EV-F showed the upregulation of SD-S67 VP1 protein and mRNA expression as well as TCID_50_ (Fig. 8K through N).

Taken together, these results demonstrated that GRP78 inhibits virus replication by facilitating the activation of NF-κB and triggering the expression of proinflammatory factors.

## DISCUSSION

In this study, we investigated the functions of a novel identified EV 3D-interacting protein, GRP78, and demonstrated that GRP78 interacts with EV-encoded 3D protein and the NF-κB signaling proteins CHUK and IKBKB to facilitate the activation of the NF-κB signaling pathway and inhibit the enterovirus replication via triggering the expression of proinflammatory factors. Our findings revealed a new role of GRP78 in regulating host innate immunity in response to viral infection and provide new insights into the mechanism underlying enterovirus replication and NF-κB activation.

GRP78, a member of the heat shock protein 70 kDa family, plays a critical role in maintaining endoplasmic reticulum homeostasis and functions as the molecular chaperones for protein folding by binding to the misfolded proteins or unassembled complexes to initiate the ER-associated degradation responsible for unfolded protein response (UPR) regulation (48, 49). Nakajima et al. demonstrated that GRP78 was selectively cleaved by subtilase cytotoxin (SubAB) to trigger ER stress condition, where SubAB preferentially induce C/EBPβ through the ATF6 branch of the UPR, leading to the interaction of C/EBPβ with p65 and the inhibition of NF-κB signaling pathway activation. SubAB also use another strategy to repress the NF-κB pathway by phosphorylation of mTOR via activating the ATF6 pathway and downregulating Akt phosphorylation (50). Sunitinib, a drug for the treatment of renal cell carcinoma, activates the NF-κB signaling pathway through the IRE1α/TRAF2/IKKβ axis of the ER stress response, enhancing the expression of IL-6, IL-8, and TNF-α. Sunitinib also activates PERK to induce the expression of cytokines IL-6, IL-8, and TNF-α (51). Crane et al. reported that ER stress promotes the repositioning of GRP78 to the cell surface (csGRP78), which displays antigenic properties and leads to the production of anti-GRP78 autoantibodies. The binding of anti-GRP78 autoantibodies to csGRP78 on human endothelial cells activates NF-κB, thereby inducing the expression of adhesion molecules ICAM-1 and VCAM-1 in human endothelial cells (52). Three branches of UPR (IRE1α, PERK, and ATF6) of ER stress are reported to cross-talk with the NF-κB pathway. For example, reactive oxygen species (ROS) causes ER stress, activating downstream IRE1α, subsequently recruiting TRAF2 to activate the JNK1/2 and NF-κB signaling pathways (53). IRE1α splitting viral mRNA activates RIDD/RIG-I, causing cellular autoimmune inflammation through the NF-κB and IFN pathways (54). Zhang et al. observed that anti-dsDNA antibodies induce NF-κB activation and upregulated the expression of IL-1β, TNF-α, and MCP-1 through PERK-eIF2α-ATF4 (55). In addition, GRP78 functions as a co-receptor for several viruses such as Coxsackievirus A9, Borna disease virus, and Zika virus (16–19). Although GRP78 has been demonstrated to bind to some viral structural proteins involved in the degradation of viral misfolded proteins during protein production as well as in virus assembly (56–58), little was known about the role of GRP78 in host innate immune response against pathogen infection. We employed a previously identified EV-F SD-S67 strain to explore the function of GRP78 and found that GRP78 expression was significantly increased in cells infected by enterovirus. Moreover, the expression of GRP78 was demonstrated to be in a time- and dose-dependent manner and a cell line-independent fashion. Similar findings were also observed in cell lines infected by EV-E HY12 strain (data not shown), indicating a general expression pattern for GRP78 upon enterovirus infection. It is interesting to note that the overexpression of GRP78 inhibits virus replication, while the knockdown of GRP78 promotes virus replication, suggesting that GRP78 was indeed involved in the regulation of enterovirus replication. The co-immunoprecipitation assay and confocal microscopy revealed that only 3D interacted with GRP78 and ruled out the interaction between GRP78 and other EV-encoded proteins. 3D protein is an EV-encoded RdRP involved in viral genome synthesis during the virus replication cycle. The findings that GRP78 specifically interacts with 3D protein indicates it is likely one of the components for the 3D protein complexes involved in viral RNA synthesis and virus replication, which is a subject for future investigation.

NF-κB is highly activated in diverse virus infections, including the EV71 (59), Coxsackievirus B3 (60), and foot-and-mouth disease virus (61). NF-κB-induced inflammation plays an important role in pathogenesis and disease development. Previous studies showed that bovine enterovirus infection increased the expression levels of proinflammatory cytokines, including phosphorylation levels of JNK/SAPK, p38 MAPK proteins, and IL-6 (62). In this study, we demonstrated that EV-F infection activates NF-κB in 293T cells and Raw264.7 cells using a luciferase reporter gene assay and IFA, and the expression of proinflammatory genes induced by EV-F SD-S67 infection was associated with NF-κB activation. The finding that treatment with the NF-κB-specific inhibitor significantly decreased EV-F SD-S67-induced proinflammatory cytokine production suggested that inflammation during EV-F SD-S67 infection is due to NF-κB activation.

Emerging evidences indicated that NF-κB signaling is essential for EV71 replication and EV71-induced inflammatory responses (63, 64). Many EV-encoded proteins have been identified to play a critical role in the regulation of inflammation. EV71 2C protein inhibits NF-κB activation by either binding to the IPT domain of p65 and reducing the formation of heterodimer p65/P50 or through association with IKKβ (8). Enteroviruses inhibit IKKβ phosphorylation by recruiting PP1 and IKKβ to form a complex through 2C proteins, which ultimately results in the inhibition of the NF-κB signaling pathway (7). EV71 infection activates NF-κB signaling and further regulates multiple inflammatory cytokines in different cell types (63–65). EV 3D protein is an RNA-dependent RNA polymerase and is responsible for the genomic replication of enterovirus. A previous study has clarified that EV71-activated SIRT1 binds with the 3D protein and attenuates the acetylation and RdRp activity of 3D, resulting in the repression of viral genome replication (66). Some studies have also reported other functions of 3D; one research study reported that EV71 mediated cell cycle arrest in S phase through 3D (67). Wang et al. demonstrated that EV71 3D promotes the activation of the NLRP3 inflammasome through binding to NLRP3 (68). Recently, a new finding pointed out that EV71 3D inhibits MDA5-mediated IFN-β promoter activation via interacting with the CARD domain of MDA5 protein (69), and another claimed that beclin1 binds to EV71 3D protein to promote virus replication (70). In the current study, we screened the EV-F-encoded proteins, demonstrated that 3D protein was the most potent NF-κB activator, and found that the proinflammatory IL-1β, IL-6, IL-8, and TNF-α production is induced mainly by the activation of the NF-κB signaling pathway. In addition to 3D, our results showed that VP3, 2A, and 3C were also potential NF-κB activators. The functions of these potential NF-κB activators in the regulation on virus replication need to be investigated in the future.

Although we have demonstrated that 3D protein binds to GRP78 and affects EV-F SD-S67 infection, further research is required to determine whether this interaction is involved in host innate immune response. Several studies have demonstrated the interactions and regulation of NF-κB with Hsp members, where GRP78 is a member of the same Hsp protein family (24, 28). The interaction interface of the docking model showed the key residues between GRP78 and NF-κB involved in the docking (71). In this study, we demonstrated that GRP78 is involved in the NF-κB signaling pathway by employing the GRP78 knockdown and overexpression cell lines. We found that GRP78 has a significant effect on the host innate immune responses of NF-κB and contributes to the inhibition of EV-F SD-S67 replication. More importantly, we demonstrated that GRP78 directly interacts with the NF-κB signaling proteins CHUK and IKBKB to activate NF-κB signaling, leading to the expression of the proinflammatory factors and inhibition of virus replication.

**Fig 9:**
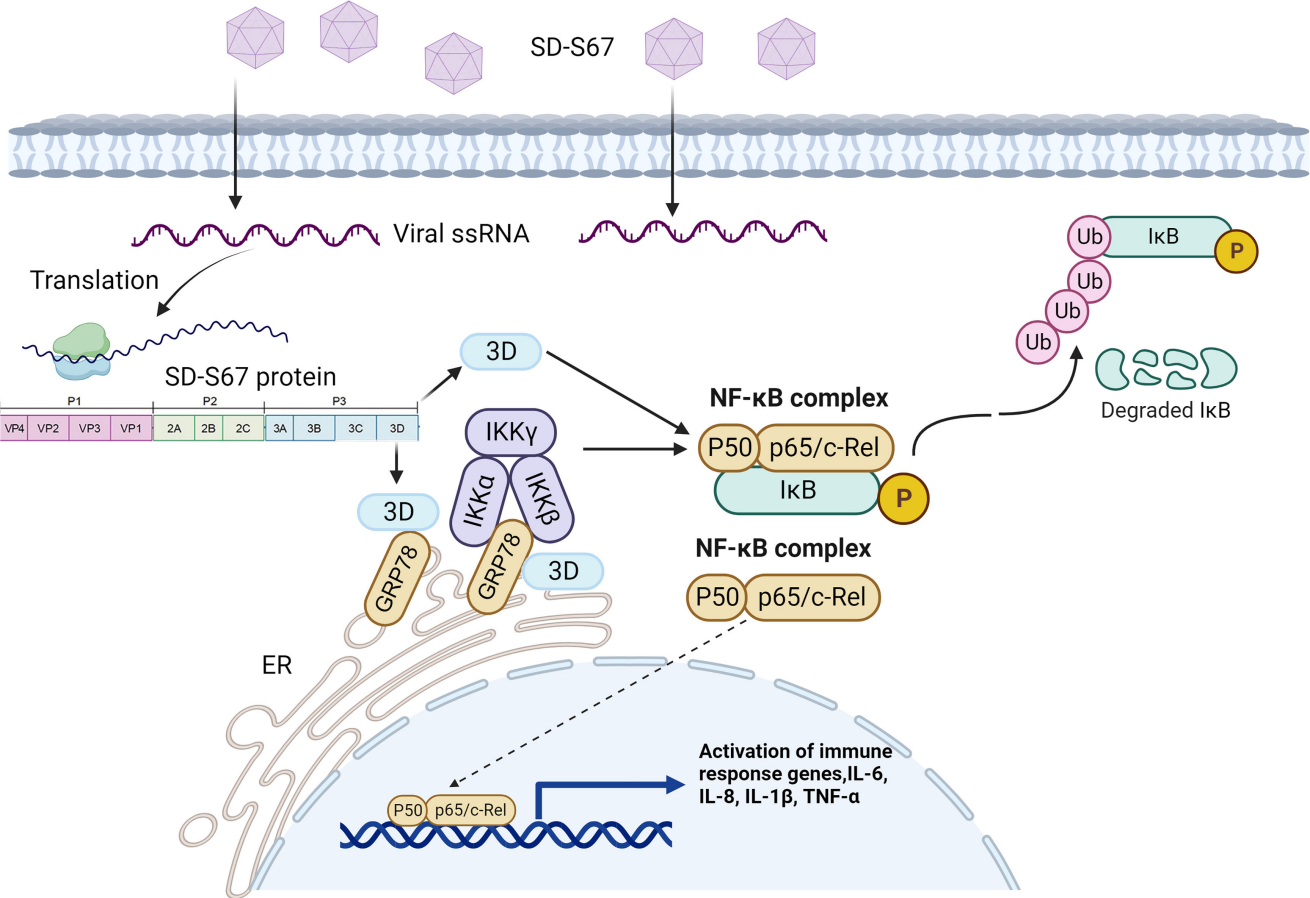
A proposed preliminary model for inhibiting EV replication by GRP78. Upon EV-F SD-S67 infection, GRP78 was significantly induced. The induced GRP78 interacts with EV-encoded 3D RNA polymerase and the NF-κB upstream signaling molecules CHUK/IKBKB to form a potential “3D-GRP78-CHUK/IKBKB” protein complex, which facilitates the phosphorylation and nuclear translocation of NF-κB, activates the NF-κB signaling pathway, and triggers the increasing production of inflammatory cytokines such as IL-6, IL-8, IL-1β, and TNF-α, thus leading to the inhibition of enterovirus replication.

Based on the results, we proposed a preliminary model for the regulation of GRP78 on EV-F SD-S67 replication (Fig. 9). After EV infection, the GRP78 was increased and interacts with the viral 3D RNA polymerase and the NF-κB signaling proteins CHUK and IKBKB to activate NF-κB by forming a “3D-GRP78-CHUK/IKBKB” structure. The binding of CHUK/IKBKB with GRP78 facilitates the phosphorylation and nuclear translocation of NF-κB and increases the production of inflammatory cytokines, such as IL-6, IL-8, IL-1β, and TNF-α, thus leading to the inhibition of virus replication.

In conclusion, we discovered a new role of 3D and GRP78 as an important regulator in the activation of inflammatory response and revealed a novel mechanism underlying NF-κB activation by GRP78, which provides new insights into the development of an agent for the treatment and prevention of viral associated inflammation and diseases.

## MATERIALS AND METHODS

### Cell cultures and virus

Vero cells and 293T cells were cultured in Dulbecco’s modified Eagle’s medium (Invitrogen, Carlsbad, CA, USA) supplemented with 5% fetal bovine serum (HyClone, Beijing, China), 2 µg/mL gentamycin, and 2 mM L-glutamine (Invitrogen). Raw264.7 cells were cultured in Roswell Park Memorial Institute-1640 (Invitrogen) supplemented with 10% fetal bovine serum (HyClone), 2 µg/mL gentamycin, and 2 mM L-glutamine (Invitrogen). The enterovirus SD-S67 strains (GenBank accession no. MK639928) were isolated and kept in our laboratory (37, 46). The propagation and titration of SD-S67 were performed using Vero cells. Virus titration was performed using Vero cells in 96-well plates and expressed as the TCID_50_ per unit volume as described previously (72).

### Antibodies

Rabbit anti-β-tubulin pAbs (AC008), rabbit anti-GRP78/BiP pAb (A11366), rabbit anti-His pAb (AE068), rabbit anti-Flag pAb (AE063), mouse anti-Flag pAb (AE005), mouse anti-HA monoclonal antibodies (mAbs) (AE008), rabbit anti-p65 mAb (A19653), rabbit anti-phospho-NF-κB p65 mAb (AP1294), and rabbit anti-IKBα mAb (A19714) were purchased from ABclonal Technology (Wuhan, China). Goat anti-mouse IgG (H&L)-Alexa Fluor 594 secondary antibody (RS3608), DyLight 488-goat anti-rabbit secondary antibody (RS23220), horseradish peroxidase (HRP)-conjugated goat anti-rabbit IgG antibody (RS0002), and HRP-conjugated goat anti-mouse IgG antibody (RS0001) were purchased from Immunoway (California, USA). Rabbit anti-SD-S67-VP1 pAb, mouse anti-SD-S67-VP1 pAb, and mouse anti-SD-S67-3D pAb were generated from our laboratory.

### Reagents

Polyethylenimine (PEI) (24765) transfection reagent was purchased from Polysciences (Warrington, PA, USA). ABScript III RT Master Mix for qPCR with gDNA Remover (RK20429) and 2× Universal SYBR Green Fast qPCR Mix (RK21203) were purchased from ABclonal Technology (Wuhan, China). RNAiso Plus (9109) was purchased from Taraka (Kyoto, Japan). Mouse anti-Flag Magnetic Bead (YJ007), mouse anti-His Magnetic Bead (YJ009), and RIPA lysis buffer were purchased from Epizyme Technology (Shanghai, China). Anti-HA Nanobody Magarose Beads (KTSM1335) was purchased from AlpalifeBio Technology (Shenzhen, China). NF-κB inhibitor Bay 11-7082, Dual Luciferase Reporter Gene Assay Kit, and Calcein AM Cell Viability Assay Kit (CCK-F) were purchased from Beyotime Biotechnology (Shanghai, China).

### Plasmid constructs and transfection

The plasmids expressing the SD-S67-encoded proteins and EV71-3D protein were constructed by cloning the RT-PCR-amplified corresponding fragments from EV strain SD-S67 (GenBank accession number: MK639928) and EV71 strain (GenBank accession number: HQ611148.1) to the pCMV-Tag2B vector (Flag-tagged-encoded protein). The fragments of SD-S67 VP3 and VP4 were cloned into pEGFP-N1 (GFP-tagged VP3 and GFP-tagged VP4). The primers used in vector construction are shown in Table 1. The full-length GRP78 gene (GenBank: NM_005347) was amplified from 293T cells and inserted into the *Bam*H I and *Eco*R I sites of pLV3-CMV-CopGFP-Puro vector (Invitrogen) to generate pLV3-CMV-GRP78-6×His-CopGFP-Puro (His-tagged GRP78). The full-length CHUK gene (GenBank: NM_001278) amplified from 293T cells was inserted into the *Hind* III and *Xho* I sites of pCDNA3.1 vector (Invitrogen) to generate pCDNA3.1-CHUK-3×HA (HA-tagged CHUK). The full-length IKBKB gene (GenBank: NM_001190720) was amplified from 293T cells and cloned into the *Bam*H I and *Eco*R I sites of pEnCMV vector (Invitrogen) to generate pEnCMV-IKBKB-3×FLAG (Flag-tagged IKBKB). The luciferase reporter plasmid pNF-κB-Luc containing κB binding motifs and the luciferase reporter gene (Luc) and the control plasmid pRL-SV40 were purchased from Beyotime Biotechnology (Shanghai, China).

**TABLE 1:** Primers used for vector construction^*a*^

Gene name	Forward primer sequence (5′−3′)	Reverse primer sequence (5′−3′)
VP1	**CGCGGATCC**GCCACCATGGGGGATGTTAAGGACTCAA	**CCGGAATTC**AGCAGTGGTGATGGAGCTG
VP2	**CGCGGATCC**GCCACCATGAGCCCGAGTGCTGAAGCGTG	**CCGGAATTC**CTGGGTAACTGCACGTCTGA
VP3	**CCCAAGCTT**GCCACCATGGGACTCCCCACAATGTACA	**CCGGAATTC**CTGGAGGGCTGCGGTCTGA
VP4	**CCCAAGCTT**GCCACCATGGGTGCGCAAGTAAGCA	**GGGTACC**TCTTTCAGGGGGACTGCGGCCTCC
2A	**CGCGGATCC**GCCACCATGGGAGCATTTGGCCAACAGAGT	**CCGGAATTC**CTGCTCCATCACATCATCC
2B	**CGCGGATCC**GCCACCATGGGATTGACCGATTATGTG	**CCGGAATTC**CTGCTTCTGAGCCATGTT
2C	**CGCGGATCC**GCCACCATGGCAGATAGTTGGATTAAGA	**CCGGAATTC**TTGGAAGAGCGCTTCAAT
3A	**CGCGGATCC**GCCACCATGGGACCTCCTGAGTTTAAG	**CCGGAATTC**TTGCAGTCCAGCAAAGAG
3C	**CGCGGATCC**GCCACCATGGGACCCCTCCTTGATTTCG	**CCGGAATTC**TTGTGGTTGCGTGAAGTA
3D	**CGCGGATCC**GCCACCATGGGGGAGATTGAATTCATGGA	**CCGCTCGAG**GAAGGAGTCGTACCAGGAGC
EV71-3D	**CGCGGATCC**GCCACCATGGGAGAGATCCAGTGGGTTAA	**CCGGAATTC**AAATAACTCGAGCCAATTGCGTC
GRP78	**CCGGAATTC**GCCACCATGAAGCTCTCCCTGGTGG	**CGCGGATCC**CAACTCATCTTTTTCTGCTG
CHUK	**CCCAAGCTT**GCCACCATGGAGCGGCCCCCGGGGCTG	**CCGCTCGAG**TTCTGTTAACCAACTCCAAT
IKBKB	**CGCGGATCC**GCCACCATGAGCTGGTCACCTTCCCT	**CCGGAATTC**TGAGGCCTGCTCCAGGCAGC

^*a*^ The restriction enzyme cutting sites are highlighted in bold.

For the transfection experiments, the corresponding plasmids were transfected using PEI (Polysciences) according to the manufacturer’s instructions.

### Reporter gene assays

The firefly luciferase and Renilla luciferase activities were detected using the Dual Luciferase Reporter Gene Assay Kit (Beyotime Biotechnology, China), according to the manufacturer’s protocol. Cell viability assays were carried out using the Calcein AM Cell Viability Assay Kit (CCK-F) (Beyotime) following the manufacturer’s instructions. All reporter gene assays were repeated at least three times. Data are presented as the mean ± SD values.

### GRP78 knockdown using lentivirus infection

Knockdown of GRP78 was performed using the lentivirus generated following the manufacturer’s instruction. The shRNA sequences for targeting the human GRP78 gene were designed and used to generate pLVX-shGRP78-a and pLVX-shGRP78-b plasmids as follows: sh-GRP78-a: 5′-TGGCCTAAATGTTATGAGGATCTTCAAGAGATCCTCATAACATTTAGGCCA-3′; sh-GRP78-b: 5′-AGATTCAGCAACTGGTTAAAGTTCAAGAGACTTTAACCAGTTGCTGAATCT-3′. The generated pLVX-shGRP78-a or pLVX-shGRP78-b plasmid was co-transfected into 293T cells with psPAX2 and pMD2G in the ratio 4:4:1 using PEI (Polysciences). The packaged lentiviruses harvested at 48 and 72 h post-transfection were used to infect the 293T cells in the presence of 4 µg/mL polybrene (Sigma). Forty-eight hours post-infection, the cells were selected using 1.5 µg/mL puromycin (Sigma). The resulting cells were harvested to examine the expression of GRP78 and the targeting genes using qPCR and immunoblot assay. The pLVX vector was used as a negative control (Beyotime, China).

### IFA and confocal microscopy

Vero cells or 293T cells were prepared on 20 mm covered glass bottom dishes. The cells were fixed and permeabilized with ice-cold methanol for 20 min. After blocking with 5% skim milk for 1 h, the cells were incubated with indicated primary antibodies for 1 h, followed by probing with Alexa Fluor 594-labeled goat anti-mouse IgG and/or DyLight 488-labeled goat anti-rabbit IgG antibodies. Cell nuclei were stained with 1 µg/mL DAPI (Roche) methanol solution. After mounting with Prolong Glass Antifade Mountant (Thermo Fisher Scientific), the samples were visualized by confocal laser scanning microscopy (Fluoview FV3000; Olympus).

### Immunoprecipitation and immunoblot analysis

Cells were harvested, and whole-cell lysates were prepared on ice by lysing cells with RIPA lysis buffer containing 10% protease inhibitor cocktail (Epizyme, China). The cell lysates were sonicated and then centrifuged at 12,000 rpm at 4°C for 20 min. Lysates were successively immunoprecipitated with mouse anti-Flag Magnetic Beads, mouse anti-His Magnetic Beads, or anti-HA Nanobody Magarose Beads overnight at 4°C.

The protein concentration of each sample was determined by the BCA protein assay kit (Epizyme, China). An equal number of total proteins was loaded, separated by SDS-PAGE, and normalized by β-tubulin. After transferring onto the polyvinylidene fluoride membranes (Millipore, Massachusetts, USA), the membranes were blocked in 3% skim milk and incubated with the primary antibodies before probing with the corresponding secondary antibodies (conjugated with HRP). After washing, the ECL reagent (Yeasen, China) was used for the chemiluminescent detection of target proteins following the manufacturer’s instructions. The protein band was visualized using a Luminescent Image Analyzer (Tanon 4600SF, Shanghai).

### Real-time reverse transcription PCR

Total RNAs were extracted using a RNAiso Plus reagent (Takara, Japan) and reversely transcribed into cDNAs with ABScript III RT Master Mix for qPCR with gDNA Remover (ABclonal Technology, China). The cDNAs from different samples were amplified by PCR using a 2× Universal SYBR Green Fast qPCR Mix (ABclonal Technology, China). The relative mRNA abundance was normalized to the expression of GAPDH and evaluated by the 2^-ΔΔCT^ method (73). Primers for real-time PCR were designed by Primer-BLAST, and their sequences are in Table 2.

**TABLE 2:** Primers used for qPCR^*a*^

Gene name	Forward primer sequence (5′−3′)	Reverse primer sequence (5′−3′)	
m-GAPDH	AGGTCGGTGTGAACGGATTTG	GGGGTCGTTGATGGCAACA	
m-IL-6	CCGGAGAGGAGACTTCAC	TCCACGATTTCCCAGAGA	
m-IL-8	AACCTAGGCATCTTCGTCCG	ATTGGGCCAACAGTAGCCTT	
m-IL-1β	TTGAAGAAGAGCCCATCCTC	CAGCTCATATGGGTCCGAC	
m-TNF-α	TAGCCAGGAGGGAGAACAGA	TTTTCTGGAGGGAGATGTGG	
h-GAPDH	AAGGCTGTGGGCAAGG	TGGAGGAGTGGGTGTCG	
h-IL-6	GTACATCCTCGACGGCATCTCA	GCACAGCTCTGGCTTGTTCCTC	
h-IL-8	ACTTCTCCACAACCCTCTGC	GTTGCTCCATATCCTGTCCCT	
h-IL-1β	CACGATGCACCTGTACGATCA	AGCACTGAAAGCATGA	
h-TNF-α	GGGTTTGCTACAACATGG	AAGAACTTAGATGTCAGTGC	
h-GRP78	GTCAGGCGATTCTGGTCATT	GGTGAAAGACCCCTGACAAA	
c-GRP78	CGGAGGAGGAGGACAAGAAGGAG	ATAAGACGGCGTGATGCGGTTG	
c-GAPDH	AAGTTCAACGGCACAGTCAAGG	CACATACTCAGCACCAGCATCAC	
SD-S67-VP1	GGACTCAATCAAGGGGGCAG	CTGAGGTTTCCTCGACACCG	

^*a*^ M represents mouse, h represents human, and c represents cattle.

### Statistical analysis

All experiments were repeated at least three times. Statistical significance for comparison of two means was assessed by an unpaired Student t-test. For dose-dependent experiments or multiple comparisons, a one-way ANOVA test was used followed by a *post hoc* test (Dunnett or Tukey test). Analyses were performed using the Prism 9 software (GraphPad). Means were illustrated using a histogram with error bars representing ±SD, and statistical relevance was evaluated using the following *P*-values: ^*^P < 0.05, ^**^P < 0.01, and ^***^P < 0.001.

## Data Availability

The authors state that all data related to this study are available. The enterovirus strain used in this study is SD-S67. The GenBank accession number is MK639928.1.
